# Phylodynamic Reconstruction Reveals Norovirus GII.4 Epidemic Expansions and their Molecular Determinants

**DOI:** 10.1371/journal.ppat.1000884

**Published:** 2010-05-06

**Authors:** J. Joukje Siebenga, Philippe Lemey, Sergei L. Kosakovsky Pond, Andrew Rambaut, Harry Vennema, Marion Koopmans

**Affiliations:** 1 National Institute for Public Health and the Environment, RIVM, Bilthoven, The Netherlands; 2 Erasmus Medical Centre, Rotterdam, The Netherlands; 3 Rega Institute, Katholieke Universiteit Leuven, Leuven, Belgium; 4 Antiviral Research Center, University of California San Diego, San Diego, California, United States of America; 5 Institute of Evolutionary Biology, University of Edinburgh, Edinburgh, United Kingdom, and Fogarty International Center, National Institutes of Health, Bethesda, Maryland, United States of America; Cornell University, United States of America

## Abstract

Noroviruses are the most common cause of viral gastroenteritis. An increase in the number of globally reported norovirus outbreaks was seen the past decade, especially for outbreaks caused by successive genogroup II genotype 4 (GII.4) variants. Whether this observed increase was due to an upswing in the number of infections, or to a surveillance artifact caused by heightened awareness and concomitant improved reporting, remained unclear. Therefore, we set out to study the population structure and changes thereof of GII.4 strains detected through systematic outbreak surveillance since the early 1990s. We collected 1383 partial polymerase and 194 full capsid GII.4 sequences. A Bayesian MCMC coalescent analysis revealed an increase in the number of GII.4 infections during the last decade. The GII.4 strains included in our analyses evolved at a rate of 4.3–9.0×10^−3^ mutations per site per year, and share a most recent common ancestor in the early 1980s. Determinants of adaptation in the capsid protein were studied using different maximum likelihood approaches to identify sites subject to diversifying or directional selection and sites that co-evolved. While a number of the computationally determined adaptively evolving sites were on the surface of the capsid and possible subject to immune selection, we also detected sites that were subject to constrained or compensatory evolution due to secondary RNA structures, relevant in virus-replication. We highlight codons that may prove useful in identifying emerging novel variants, and, using these, indicate that the novel 2008 variant is more likely to cause a future epidemic than the 2007 variant. While norovirus infections are generally mild and self-limiting, more severe outcomes of infection frequently occur in elderly and immunocompromized people, and no treatment is available. The observed pattern of continually emerging novel variants of GII.4, causing elevated numbers of infections, is therefore a cause for concern.

## Author Summary

Noroviruses, known as the viruses that cause the ‘stomach flu’ or as the ‘cruise ship virus’, cause sporadic cases and large outbreaks of gastrointestinal illness in humans. An increase in norovirus outbreaks was reported globally around 2002. Doubts remained as to whether this increase was real, or caused by improved detection-techniques and increased awareness. This study was performed to address this ambiguity, and to determine the possible virological causes for such changes. Using a population genetic approach, we studied sequences of epidemic norovirus strains collected through time and we indeed demonstrated expanding epidemic dynamics. Global epidemics were caused by subsequent variants of norovirus, observed in 2002, 2004 and 2006 and at a smaller scale in 1996, whereas no evidence for such epidemic evolutionary patterns occurring previous to these peaks. Based on the sequences analyzed the strains of the genotype under study here were shown to have circulated at least since the early 1980s, and likely earlier. We showed that not only surface exposed sites on the outside of the virus shell were under selective pressure, involved in avoiding host immune responses, but also codons that are apparently conserved for the purpose of virus replication.

## Introduction

Noroviruses (NoV) are the most common cause of acute viral gastroenteritis [1], [2], with the numbers of reported outbreaks peaking characteristically between November and March in the northern hemisphere [3]. Illness is usually self-limiting and symptoms, comprising acute onset vomiting and watery diarrhea, subside within one to three days [4]. The relevance of studying NoV lies in their high prevalence in the population [5], and in the more severe and prolonged illness that is seen among elderly and immunosuppressed patients [6]–[8]. NoVs are highly infectious, due to the combination of an extremely low infectious dose (an estimated ID50 of less than 20 viral particles [9]), very high levels of shedding (around 10^8^ but up to >10^10^ RNA copies per gram of stool) and prolonged shedding after clinical recovery [10], [11]. NoV outbreaks, which may affect hundreds of people and are notoriously difficult to control, are primarily associated with places where people are in close contact, for example hospitals and long-term care facilities.

NoVs are a genetically diverse group of positive sense single-stranded RNA viruses from the *Caliciviridae* family. Their 7.5 kb genome includes three open reading frames (ORFs). The first ORF encodes a polyprotein that is post-translationally processed to form the non-structural proteins, the second and third ORFs encode the major and minor structural proteins; VP1 or the capsid protein and VP2. The viral capsid is formed by 180 copies of the major capsid protein, and governs antigenicity, host-specificity and environmental stability.

NoVs are classified into five distinct genogroups, which are further subdivided into genotypes, based on their amino acid capsid sequence. Molecular epidemiological studies have shown that in recent years approximately 70% of NoV outbreaks among humans have been caused by one dominant genotype, GII.4 [12]–[17].

With continuous surveillance systems in place in some countries since the mid-1990s, it has become apparent that the number of reported NoV outbreaks, and especially those caused by GII.4 strains has risen since the appearance of the 2002 variant of GII.4 [12], [15], [18]–[21]. Since then, genetically distinct GII.4 variants have emerged, and spread rapidly across the world causing epidemic waves of NoV illness [20], [22]. To date, three variants named after the year when they were first detected have been identified in populations across the world (the 2002, 2004 and 2006b variants [22]). The emergence of each of these three variants was followed by ‘hot’ NoV winters with sharply increased numbers of reported outbreaks. Older strains belonging to the lineage designated 1996 were also detected around the world, although surveillance was limited at that time.

The pattern of continuous lineage turnover, referred to as epochal evolution [23], with emerging new variants replacing previously predominant circulating ones, is strongly reminiscent of what is observed in the molecular epidemiology of Influenza A virus (IAV). The evolutionary interaction between IAV and the human immune response results in antigenic drift, illustrated by the characteristic ladder-like tree shapes for hemagglutinin and neuraminidase surface proteins [24]. Long-term partial immunity to the virus induces sharp fitness differences among strains and drives rapid amino acid replacement at key antigenic sites, pinpointed by *in vitro* and *in silico* analyses [25], [26]. Whereas antigenic data can be readily generated for IAV, allowing the comparative mapping of antigenic and genetic evolution [27], research of NoV antigenic properties has been hampered by the lack of a simple cell culture model [28]. However, recent publications indicate that the genetic differences between NoV genotypes, and also between variants of the GII.4 genotype, translate into distinct antigenic types, although molecular determinants remain largely unclear [23], [29]. Thus, individuals may be repeatedly infected by strains belonging to different genotypes, and also, because immunity against NoV infection is short-lived at best [30]–[32], possibly repeatedly by strains of the same genotype. As a result, the impact of immune responses on NoV epidemiology remains poorly understood and phylodynamic and molecular adaptation studies may provide some key insights.

In this study, we aimed to provide a rigorous measurement of NoV GII.4 diversity through time, and we investigated viral population expansions in relationship to the increased numbers of infections reported in recent years. Evolutionary and population dynamics of GII.4 NoVs were estimated by a Bayesian coalescent approach, using two different datasets of sequences from strains with known detection dates, between 1987 and 2008. One set of sequences contained full capsid sequences, the other short partial polymerase sequences, which had been obtained for standard-procedure genotyping in NoV surveillance in Europe [13] (http://www.noronet.nl/fbve/) and from the global NoV surveillance network Noronet [22] (http://www.noronet.nl/).

We also tested whether these dynamics differed from neutral expectations, so whether and how they were shaped by selective pressure, and we attempted to further elucidate the molecular determinants of NoV evolutionary and epidemiological dynamics using *in silico* techniques. To identify the molecular characteristics of NoV GII.4 strain replacement, we investigated both directional and diversifying selection and elucidated capsid protein positions showing evidence for co-evolutionary dynamics acting between sites.

## Results

### Recombination analysis

To examine the extent to which recombination has shaped NoV evolution, we analyzed an alignment of 20 GII.4 sequences, two for each variant, spanning the genome from region A (a gene region in ORF1 that is commonly used for genotyping purposes, see Materials and Methods, and [22] and [33]) up to the 3′end of the capsid. Using GARD (Genetic Algorithm for Recombination Detection), VisRD (Visual Recombination Detection) and RDP3 (Recombination detection program), significant phylogenetic variability was identified in this genome region, which could be attributed to a recombination event for the 2003Asia variant, a GII.4 variant previously identified as a recombinant lineage, mainly detected in Asia, and rarely in Europe, Oceania or the Americas [22]. The crossover point lay in the ORF1/2 overlap, a position previously identified as a recombination hotspot in NoV [34], [35]. Further analyses below were based on polymerase and capsid gene sequences that do not include this breakpoint, and no recombination could be detected in those individual data sets using the Phi test [36].

### Time-measured phylodynamic analyses

To test whether GII.4 evolution deviated from selective neutrality, we applied a genealogical neutrality test that involved Bayesian coalescent inference, a tree-based summary statistic (*D_F_*), and posterior predictive simulation [37]. Using a constant population size demographic prior, the capsid data set (194 sequences, 1623 nt) showed significantly more negative *D_F_* than expected (P<0.01), suggesting a selective process that generated a significant excess of mutations on terminal branches. The same was true using an exponential growth prior, but a Bayes factor test did not support an exponential growth scenario (ln BF constant versus exponential growth = −2.47). A Bayes factor comparison also favored a constant population size model over an exponential growth model for the matched polymerase data set (172 sequences, 247 nt)(ln BF = −2.23). In this case, we did not observe a significant difference in *D_F_* (P = 0.15). Because the polymerase sequences were considerably shorter, they provided less information to evaluate branch length properties. To counteract the loss of power we increased the number of sequences, and indeed, an analysis of the complete polymerase sequences dataset (1383 sequences, 247 nt) rejected the model of neutral evolution (P = 0.001). As previously introduced [37], this neutrality test relies on relative restricted demographic models governed by a limited number of parameters to capture large-scale demographic trends. To investigate the sensitivity to demographic detail, we extended the posterior predictive simulation procedure to accommodate highly parametric demographic models, which result in a more complex picture of norovirus dynamics (see below). Using a Bayesian skyline plot (BSP) model as demographic function [38], similar conclusions could be drawn from the neutrality test: significantly more negative *D_F_* values than expected under neutrality were observed for the capsid data set (P = 0.019) and the complete polymerase data set (P = 0.018), whereas the matched polymerase lacked power in rejecting neutrality (P = 0.029).

**Figure 1:**
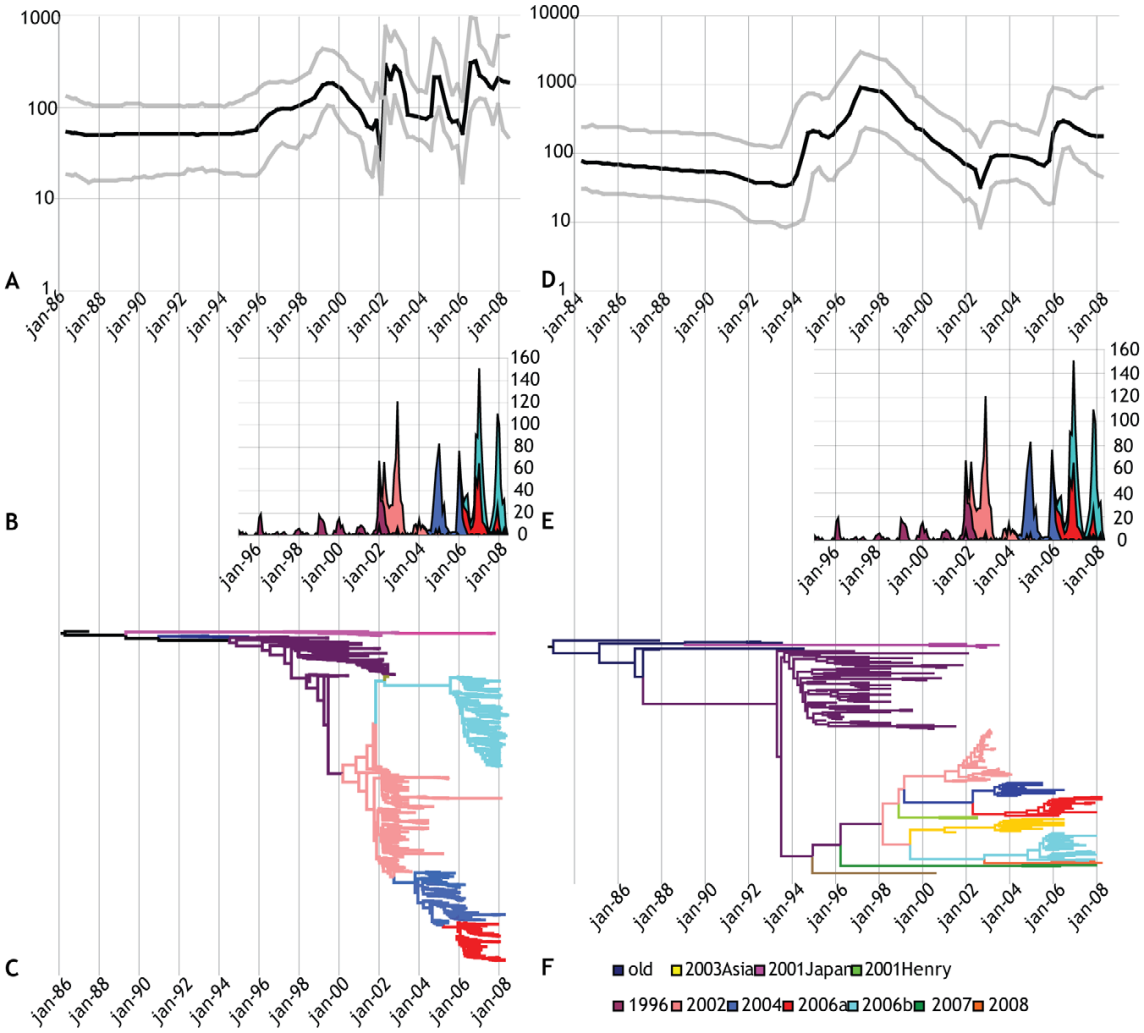
Phylodynamics of the GII.4 noroviruses. The left panel (A, B, C) describes analysis of the polymerase dataset, the right panel (D, E, F) the analysis of the capsid dataset. A) Bayesian Skyline Plot (BSP) of GII.4 NoV partial polymerase sequences, representing the relative genetic diversity, a measure for the number of effective infections, of circulating GII.4 NoVs through time. The black line represents the median posterior value, the grey lines the 95% Highest Probability Density (HPD) intervals. The Y-axis depicts the value of N_e_τ on a logarithmic scale. B and E) Surveillance data of NoV GII.4 strains, detected in outbreaks, included for comparison. Only European surveillance data was obtained from the FBVE database and from Dutch surveillance [12], [13]. The different GII.4 variants detected in the outbreaks are represented by different colors, showing the displacement of subsequent variants through time. The same colors were used in panels D and F, the color legend is shown under panel F. C) Maximum Clade Credibility (MCC) Tree of NoV GII.4 partial polymerase dataset. The MCC tree represents an estimate of the posterior distribution of tree topologies and branch lengths. Different variants are represented in different colors, the tree is scaled in units of time with tips constrained to strain detection dates. D) BSP of the GII.4 NoV capsid dataset. F) MCC tree of the GII.4 NoV capsid dataset.

The demographic inference using the BSP model is summarized in Figures 1A and 1B, which essentially plot N_e_τ as a function of time. N_e_ τ can be considered a measure of relative genetic diversity that, in turn, reflects the number of effective infections established by the virus (see also the Materials and Methods section). Uncertainty in the estimated parameters was evaluated using 95% Highest Probability Density (HPD) intervals. The Maximum Clade Credibility (MCC) trees from the same Bayesian analyses (Figures 1C, D) summarize the NoV evolutionary histories, and the stepwise emergence of the subsequent variants on a time scale. For comparison, surveillance data of reported NoV outbreaks with confirmed GII.4 variant type were imposed on the BSPs.

The changing patterns of NoV genetic diversity generally revealed seasonal dynamics, albeit with markedly varying resolution among the two datasets. The BSP for the polymerase dataset (Figure 1A) showed peaks for N_e_τ that coincided with the epidemic peaks observed in norovirus surveillance systems in the northern hemisphere winters 2002–03, 2004–05 and 2006–07. The BSP obtained from the capsid dataset (Figure 1B) showed a pattern that was more difficult to reconcile with epidemiological observations. Values for N_e_τ were highest in the years 1997–1999, and the emergence of the 2002 variant, which had a strong impact in the population according to surveillance data, did not coincide with a pronounced upsurge in the BSP. Comparison of the BSPs obtained for both genes, illustrated that unraveling seasonal population dynamics with associated population bottlenecks for viruses like NoV, may require a sufficiently high sampling density. In fact, reducing the partial polymerase dataset to a similar number of sequences drastically diminished the resolution of the BSP analysis (Figure S1). In particular the 2004–05 and 2006–07 epidemics were not well reflected in the BSP derived from this sub-set of polymerase sequences that matched the capsid dataset in size, both genetically and temporally. The 2002–03 epidemic, following the replacement of the 1996 variant by the 2002 variant, wás however clearly noticeable in the matched polymerase set, whereas it was not in the capsid data set. Considering the associated MCC trees (Figures 1C, 1D), it is conceivable that following the relatively long build-up of genetic variation during the circulation of the 1996-variant, its replacement by the 2002 variant signified a massive and sudden loss of diversity; a population bottleneck. The 2002 variant split into two distinct subclusters for the polymerase dataset. These lineages arose almost immediately after the emergence of the 2002 variant, and individually coalesced to a Most Recent Common Ancestor (MRCA) shortly before their diversification. The capsid 2002 variant cluster also grouped in two sublineages, but they coalesced more gradually to their MRCA.

**Figure 2:**
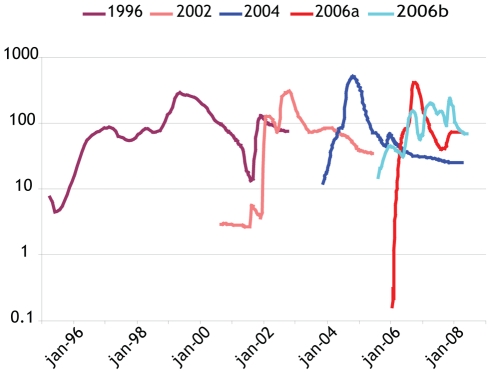
Bayesian Skyline Plots of different GII.4 variants analyzed separately. Only the mean population sizes through time were plotted for clarity.

Comparison of the variant dynamics in the MCC trees to their respective BSPs suggested that variant replacement was not always absolute across subsequent epidemic seasons. To investigate this in more detail, we performed the coalescent analyses on partial polymerase datasets for the individual major GII.4 variants separately (Figure 2). Whereas the pattern of rapid emergence, followed by an (epidemic) peak and later peaks of diminishing size observed for the 2002, 2004 and 2006a variants were very similar, the patterns obtained for 1996 and 2006b were quite different. The 1996 variant, that was detected in the population during a relatively long period, but at low reporting frequencies after the initial epidemic (winter of 1995–1996, in the northern hemisphere) (Figure 1A/B), showed an increasing trend in the N_e_τ values persisting long after this first peak. The 2006b strain showed a less defined pattern, with multiple, smaller peaks.

The demographic component is part of a full Bayesian model that enables the inference of time-scaled evolutionary histories and rates of molecular evolution from temporally-spaced sequence data. Rates of nucleotide substitution and the MRCA's of the included GII.4 sequences were listed in Table 1. The substitution rates found for the less densely sampled datasets, namely the complete capsid sequences (5.33×10^−3^ substitutions per site per year) and the matched polymerases set (4.32×10^−3^ substitutions per site per year) are lower than the rate found for the large partial polymerase dataset (8.98×10^−3^ substitutions per site per year). The estimated MRCA for these GII.4 strains lies in the first half of the 1980s (Table 1).

**Table 1:** Evolutionary parameters inferred by Bayesian analysis.

Dataset (number seqs, length of seq)	Median MRCA (HPD)	Median Rate (×10^−3^) Subst/site/year (HPD)
**GII.4 Capsids**	**July '82** (Oct '77–May '86)	**5.33** (4.62–6.02)
N = 194, length = 1623nt		
**GII.4 Polymerases**	**Oct '85** (Sept '82–Jun '87)	**8.98** (7.73–10.4)
N = 1383, length = 247 nt		
***GII.4 Polymerases matched to capsids***	***Jun '85*** * (Jun '79–Mar '88)*	***4.32*** * (3.50–5.20)*
*N = 172, length = 241 nt*		

### Molecular adaptation

Because the genealogical test rejected selective neutrality for the capsid gene, we attempted to identify the molecular determinants of this selective process through two different approaches that were not previously applied on NoV data, namely DEPS [38] and co-evolutionary analysis of amino acids [39], and complemented this with novel extensions of previously performed codon substitution model analyses [23], [40]. The partial polymerase sequences under analysis in this study are very short and have therefore not been included in these analyses.

### Codon substitution model

**Figure 3:**
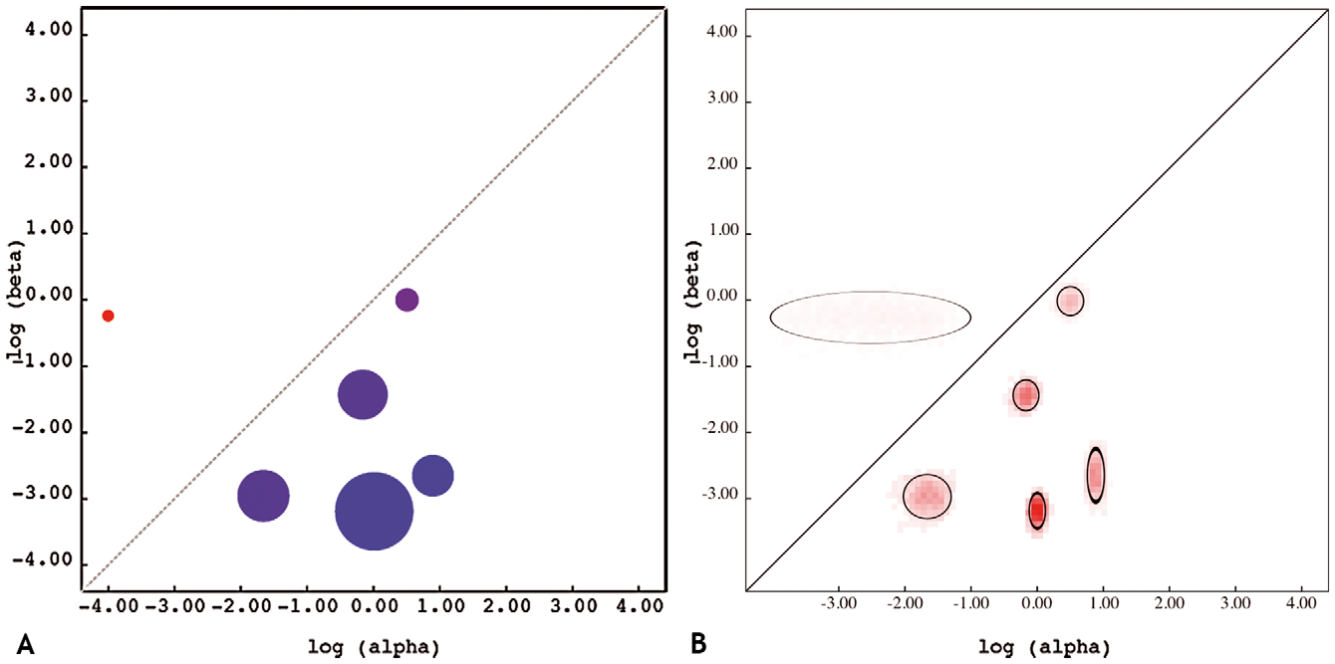
Codon substitution rate classes of GII.4 capsids. A) Inferred weights for each evolutionary rate class, showing the relative proportion of sites belonging to the given classes. The diagonal line divides the regimes of positive selection (dN/dS>1, above) and negative selection (dN/dS<1, below). B) Approximate inferred posterior distribution of synonymous (alpha) and non-synonymous (beta) substitution rates, showing the variance of each rate estimate. Color intensity is proportional to the square root of the density, and solid oval plots delineate approximate 95% confidence sets.

In order to apply a codon model based on a general bivariate discrete distribution (GBDD) of dN and dS [41], we employed a small sample AIC, which suggested that six rate classes (D = 6) provided the best fit to the capsid data. The proportion of sites within these classes and corresponding dN and dS estimates are represented by Figure 3A and 3B. The model included one rate class describing positive selection (dN ( = 0.77)>dS ( = 0.00)), with an estimated 0.93% of sites occupying this class. An empirical Bayes approach identified sites 6, 9, 15, 47 and 534 (0.93%) to be under diversifying positive selection (Table 2). Three of the sites (6, 9 and 534) were confirmed by a site-by-site Fixed Effects Likelihood (FEL) analysis at *p*<0.05, while the remaining two (15 and 47) were borderline significant (*p* = 0.06 and *p* = 0.10). The rates of false positives for FEL analyses at *p* = 0.05 was approximately 0.04 and 0.08 at *p* = 0.1, based on dataset-matched neutral simulations, suggesting that the putatively selected sites were not due to elevated rates of false positives at given nominal significance values.

**Table 2:** Codons under selection, identified by site-by-site FEL analysis.

Codon	Empirical Bayes Factor	FEL p-value	Substitution Pattern
**6**	125	0.01	0 syn
			6 non-syn (N∶S)
**9**	3125	0.007	0 syn
			9 non-syn (N∶S/T)
**15**	177	0.06	0 syn
			7 non-syn (A∶T)
**47**	37	0.10	0 syn
			6 non-syn (I∶V)
**534**	761	0.04	0 syn
			9 non-syn (A∶T/V)

To uncover population level selection processes, FEL analysis may be more appropriately applied to internal branches (iFEL) [42]. That this approach suited our data was also suggested by our genealogical tests, which identified an excess of slightly deleterious mutations on terminal branches, indicative of within host evolution. The use of iFEL enabled us to avoid this effect and revealed 8 codons under positive selection at the population level including 6, 9, 47, 352, 372, 395, 407, 534, with *p*≤0.05.

### Directional selection model

Codon models are powerful tools to detect an unusually high rate of nonsynonymous replacement, which generally occurs under a scenario of diversifying selection. However selection of episodic nature, e.g. directional selection or frequency-dependent selection is more difficult to detect and involves the question of which residues are being selected for or against [38]. A directional evolution in protein sequences analysis (DEPS) of NoV capsid sequences revealed elevated substitution rates towards 4 residues: V, S, A, T. Four sites were identified to be involved in this directional evolution; amino acids 9 (with inferred amino acid substitution pattern: N→T/S→N), 294 ((V→)A→S/P→A→T ), 333 (L→M/V/L→M→V), and 395 (-→T→A) (Figure S2).

### Epistatic effects, or co-evolutionary analyses of amino acids

**Figure 4:**
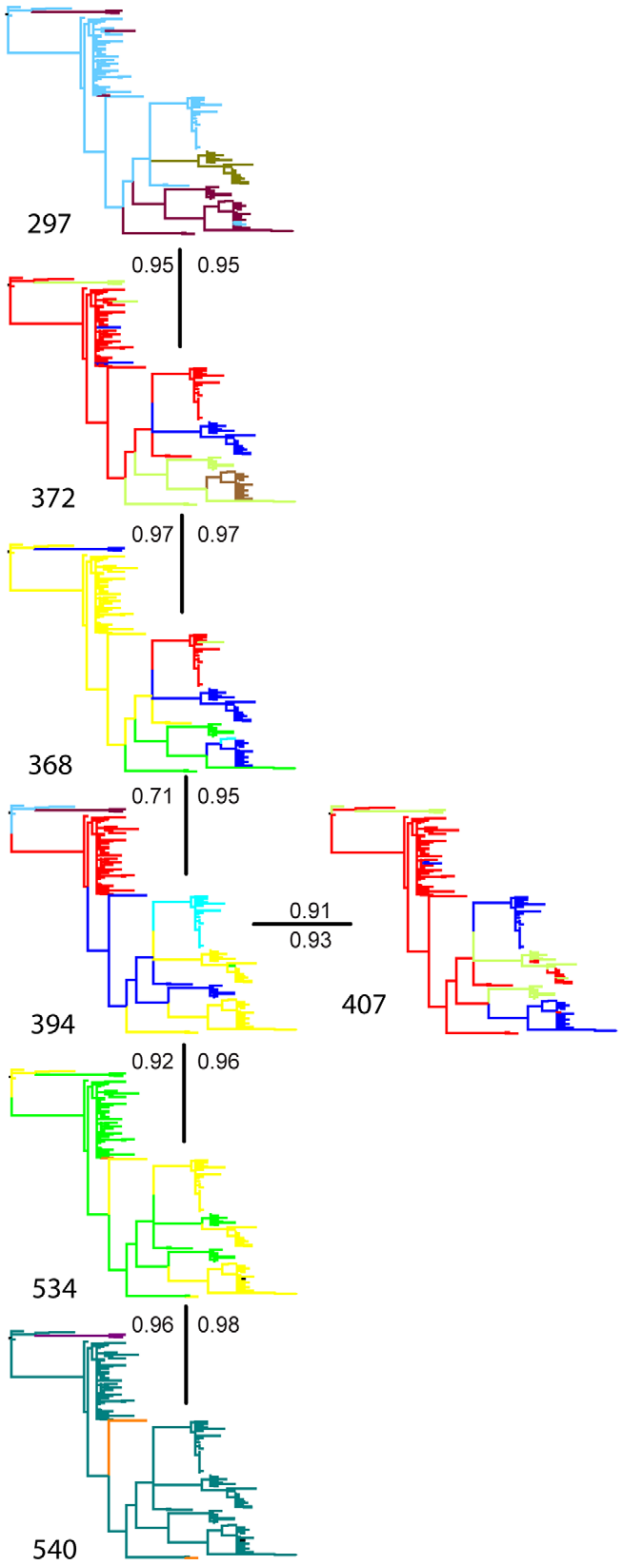
Sites identified by Bayesian graphical models to co-evolve. Amino acid sites 297, 372, 368, 394, 407, 534 and 540 of the GII.4 capsid protein were depicted as trees showing different amino acids in different colors. Each connection is associated with posterior probabilities (*P*) for single (above or left) or two (below or right) dependencies.

In folded proteins amino acids are not arranged linearly; many functionally interact, making their evolution dependant on that of others. Various types of interactions exist, and interacting sites are not necessarily direct neighbors in either the protein sequence or in the 3D protein structure. We used Bayesian graphical models (BGM) to detect co-evolving sites. The sites identified, are shown as a network in Figure 4, and sites for which co-evolution was detected but seemed less supported are shown in Figures S3A and S3B. Two values for the posterior probabilities are given, obtained from the analyses allowing for either one or two co-dependencies. Sites 231 and 209, and 238 and 504, which co-evolved as two coupled sets (Figures S3A and S3B), were not involved in recent variant transitions. Therefore we conclude that they were not under selective pressure that governed variant replacement dynamics.

**Figure 5:**
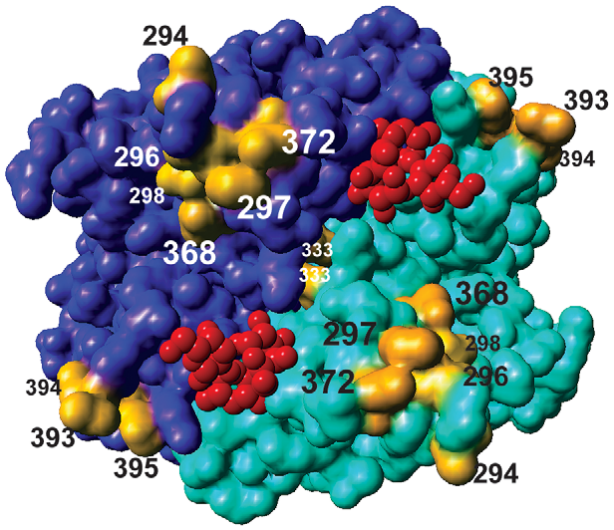
Sites identified by molecular adaptation analyses located on top of the GII.4 capsid dimer. Two parts of the dimer were given different shades of blue, the sites identified as under positive selection, co-evolving with other sites, or sites previously identified to be involved in host-interactions, were colored yellow. Ligands (B trisaccaride) bound in the binding pocket are shown in red. Protein structure 2OBT was used for generating this representation [53].

All sites for which molecular adaptation was detected are listed presented in Table 3. We marked relevant sites located on top of the capsid dimer in Figure 5.

**Table 3:** Molecular adaptation in the capsid protein, detected by different maximum likelihood approaches.

	REL	FEL	iFEL	DIR	Co-Ev
**6**	x	x	x		
**9**	x	x	x	x	
**15**	x				
**47**	x		x		
**231**					x (509)
**238**					x (504)
**294**				x	
**297**					x (372)
**333**				x	
**352**			x		
**368**					x (372, 394)
**372**			x		x (297, 368)
**394**					x (368, 407, 534)
**395**			x	x	
**407**			x		x (394)
**504**					x (238)
**509**					x (231)
**534**	x	x	x		x (394, 540)
**540**					x (534)

### RNA secondary structure predictions

**Figure 6:**
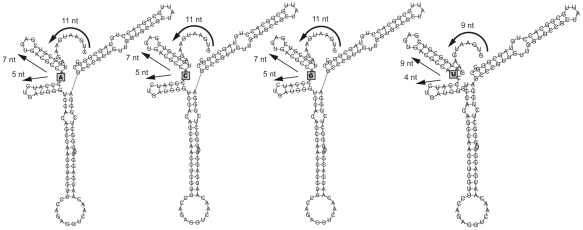
Predicted secondary structures in the 5′end of the ORF2 GII.4 RNA, with all possible nucleotides modeled at site 26 (boxed). Four nucleotides upstream of the first ATG were included, as these are conserved in the 5′end of ORF1 and ORF2 and likely to have a function in translation.

Our study identified codon 6 to be under positive, diversifying selection and codon 9 to be under diversifying as well as directional selection; others performing dN/dS analyses detected positive selection at these sites as well [23], [40]. The variation in codon 6 is due to AAT (Asn) to AGT (Ser) changes. The signal for codon 9 is solely attributable to substitutions at the second codon position of this site; AAC (Asn), ACC (Thr) and AGC (Ser). The N-terminal region of ORF2 was otherwise highly conserved. Because contrary to the other amino acids that were identified to be under selective pressure, the part of the protein encoded by these two amino acids is located on the inside of the virus capsid structure, and not surface exposed, we investigated the potential RNA secondary structure encoded by this region. *In silico* replacement of nucleotides at position 17 (codon 6) did not lead to secondary structure changes (not shown). Secondary structure predictions of the RNA encoding the 5′end of ORF2 were performed with all four possible nucleotides modeled at position 26 (Figure 6). The 4 nucleotides upstream of the ATG, generally thought to form the boundary of the subgenomic RNA [43] were included in the predictions. The presence of A, C, or G generated similar structures, when however a T (U) was modeled, extra pairing possibilities arose, lengthening the stem of the first stem-loop structure, and thus shortening the stretch of free nucleotides, available for ribosome binding, from 11 to 9.

## Discussion

Following the emergence of the genetically and antigenically novel GII.4 2002 variant in 2002, a sharp rise in the number of reported NoV outbreaks was recorded in several surveillance systems throughout the world [15], [18], [21]. Earlier, in 1995–96, a similar rise had been reported resulting from the emergence of another cluster of GII.4 - the 1996 variant [44]. The 1996 and 2002 variants have by now been succeeded by successive, highly prevalent GII.4 variants. Some uncertainty remained, however, as to whether the increase in the number of reported outbreaks was the result of a true rise in the numbers of infections, or of improved detection techniques combined with better reporting due to heightened awareness. In order to resolve this ambiguity in NoV epidemic history we applied Bayesian statistical phylodynamic techniques (Bayesian Skyline Plots, or BSPs) to alignments of a large number of GII.4 sequences. We showed that these techniques confirm the epidemic behavior of the GII.4 variants during the past decade, and that the increase in the number of NoV infections suggested from global surveillance data coincides with the rise of the GII.4 variant. Additionally we applied *in silico* techniques to identify sites under various types of selective pressure and to unravel epistatic interactions in the capsid protein.

### BSP reveals most recent epidemics, but no additional ones

The first rise in the BSP estimates of N_e_τ (a measure of effective population size) in the polymerase dataset was seen just before January 1996, around the time the first global GII.4 epidemic was noted [45] (Figure 1). Interestingly, rather than appearing as a defined epidemic peak lasting one winter season, N_e_τ increased continuously through January 2000. Surveillance data from around the world identified relatively few GII.4 outbreaks among NoV positive outbreaks in the period between 1997 and spring 2002 [12], [22]; instead, a relatively high diversity of other, non-GII.4 genotypes was detected in this period. The high N_e_τ observed for this period is congruent with the long branch-lengths in the MCC tree during this period. Given the prolonged circulation time of the 1996 variant compared to the later variants, it seems likely that the BSP in this instance is a better representative of the relatively high genetic diversity built up in an extended period of co-circulation of the 1996 variant with other genotypes, rather than of the number of infections with this particular variant. The three most recent GII.4 epidemics caused by emerging GII.4 variants were clearly visible in the polymerase BSP. The 2002 epidemic was preceded by a sharp decline in N_e_τ, indicative of a purifying selection event against the previously dominating variant, in which the diversity that had been gradually built up by the long circulation of the 1996 variant collapsed. Next, coinciding with the off-seasonal peak observed in public health surveillance systems in the northern hemisphere, N_e_τ in the BSP rose sharply. After a brief and insignificant decline another peak occurred in the winter season of 2002–03. During the epidemic caused by the 2004 variant N_e_τ peaked slightly less high and less long than during the 2002 epidemic peak. During the 2006–07 epidemic, with two distinct GII.4 variants circulating, N_e_τ was the highest (Figure 2), and after this winter N_e_τ values stayed high, corresponding with continued circulation of the 2006b variant, as also observed in surveillance.

Altogether, from the first half of the 1990s to present time, two major changes were observed. First, epidemic waves have arisen that could not be detected in earlier times, and second, the number of infections has gone up. The baseline before 1996 corresponds to the trough levels in between contemporary epidemics. Hence, the increased amount of GII.4 outbreaks observed in outbreak surveillance seems to reflect an actual increase of GII.4 infections in the population.

These conclusions are undoubtedly impacted by the comparatively few available sequences from earlier years, which are, unfortunately, not widely available, and as shown by the recent publication by Bok *et al.*, not easily attainable [40]. A search in archival stool samples revealed that during the years 1974–1981 and 1987–1991 GII.4 was not the most prevalent NoV genotype in hospitalized children with gastroenteritis, but GII.3 was. Our sampling, albeit at lower frequency during this period, would probably have detected possible unidentified epidemic surges had they occurred. Altogether, although surveillance data and demographic estimates are very different types of information, their dynamics match remarkably well. The surveillance data is presented on a linear scale and reflects the *reported* outbreaks of NoV-gastroenteritis, which is probably an incomplete description of NoV circulation, as many cases of NoV illness remain unreported. The BSPs are presented on a log-scale, which makes for less sharp peaks than the peaks observed in the surveillance data graphs.

### Different GII.4 variants reached similar levels of genetic diversity

The analysis of each major GII.4 variant separately (Figure 2) indicated that N_e_τ values, a measure for relative genetic diversity, and a proxy for the number of effective infections, of each variant reached approximately similar proportions. The 2002, 2004 and 2006a variants each had one epidemic season, but the turnover was relatively slow compared to e.g. influenza [46], resulting in repeated seasons (of decreasing magnitude) of illness caused by the same variant. For example, one main peak for N_e_τ was observed for the 2004 variant during the 2004–05 winter, but a smaller peak followed during the 2005–06 winter. This slow turnover may very well have been caused by the fact that only incomplete immunity is mounted against NoV after infection or alternatively that the pool of susceptibles is not depleted within one season. Different BSPs were obtained for GII.4 variants 1996 and 2006b, that persisted longer than one season. Interestingly N_e_τ for the 1996 variant increased just before the emergence of the 2002 variant. The 2006b variant showed no defined epidemic peak, but a series of smaller peaks that did not coincide with annual winter-peaks. Two sublineages of the 2006b variant, distinguishable by up to 5 amino acid differences in the full capsid sequence [47] circulated in the population. Of these only S368G has been recognized as a polymorphism relevant for antigenic properties. Interestingly, while only two 2006b strains with this mutation are present in our capsid dataset, later full capsid sequencing revealed more strains of this sublineage, and the polymerase dataset included more sequences from this cluster. Thus, the 2006b variant may have persisted in the population by changing its antigenic properties.

### High sampling density is necessary for accurate estimate of the effective population size of cyclic pathogen

While Bayesian coalescent analyses of the large partial polymerase dataset reflected seasonal epidemic dynamics, the analysis of the longer capsid sequences offered little detail about the phylodynamic patterns of NoV. To investigate the nature of this difference, we performed an analysis of polymerase sequences matching the capsid sequences genotypically as well as temporally, which showed that a lack of phylodynamic detail can generally be attributed to a lower sampling density (supplemental materials, Figure S1). This may not be so surprising as it was previously thought that coalescent analyses would not be so effective at capturing cyclical population dynamics [48]. Only recently, a comprehensive analysis of a large H3N2 influenza virus dataset (1302 taxa) was able to uncover seasonal dynamics from genetic data [46]. Interestingly, this data set was similar in size compared to the large dataset of partial NoV polymerases (1383 taxa) presented here, although in the influenza study full gene segments were analyzed. The population bottlenecks in the NoV GII.4 population history are to a large extent comparable to those seen for influenza and constitute repetitive large scale losses of genetic diversity. We note that viruses with seasonal dynamics do not necessarily have to exhibit such dynamics in genetic diversity. Short infections with strong cross-immunity, as seen for measles virus, allow many strains to co-circulate with frequencies contingent on neutral epidemiological processes [24]. For such viruses, seasonal epidemics may arise from repeated exhaustion of susceptible host populations [49]. Therefore, our phylodynamic analysis predicts that there may only be partial subsequent cross-immunity against GII.4 variants. It is important to note that sampling size impacts the resolution of phylodynamic inference, but the actual sampling scheme does not dictate a pattern of fluctuating population size. Rambaut *et al.* [46] performed simulations using various demographic scenarios but with a sampling scheme used to obtain influenza genetic data from seasonal epidemics. In all cases, the simulated demographic history was accurately recovered. A sparse sampling prior to 2000 also makes it difficult to unequivocally conclude an increase in GII.4 infections. However, we note that the value for N_e_τ before the first documented epidemic resulting of the emergence of a new genetic variant (the 1996-variant) in the BSP is lower than the estimates between subsequent epidemic peaks. This suggests an increase in the number of NoV GII.4 infections, which is further reinforced by a recent study of archival stool samples from the Children's Hospital, Washington, DC (1974 to 1991) (Bok et al., 2009). Although this study identified GII.4 strains in the early seventies, this was not the predominant genotype before 1991 (Bok et al., 2009). An increase of the GII.4 variant therefore seems to provide a plausible explanation for the coincident increase in the number of norovirus infections.

### Recently circulating GII.4 strains share a most recent common ancestor in the early 1980s

The estimated substitution rates (9×10^−3^substitutions per site per year for the partial polymerase sequences and 5.3×10^−3^ substitutions per site per year for the capsid sequences) corresponded to the values recently reported for NoV GII.4 [40] and were well within the range of what is commonly found for RNA viruses, e.g. between 3.5×10^−3^ and 8.5×10^−4^ for HMPV complete genomes [50] and for influenza A virus the highest rate, reported for the HA gene, was 5.72×10^−3^ substitutions per site per year [46]. A lower rate was observed for the polymerase subset matched to the capsid data set. Since the TMRCA estimates were consistent between these two polymerase data sets, the lower rate may be explained by differences in rate variation among sites, in particular for the proportion of invariant sites, which is sensitive to the number of taxa in the data set [51]. Dating the MRCA for these strains back to the early 1980's does not mean that the GII.4 lineage arose only then, but rather suggests that the strains that circulated during the past two decades share a common ancestor at that time. It seems not unlikely that the GII.4 lineage was less diverse before the 1980's, not comprising different variants as during the past decades. Alternatively, if multiple GII.4 variants did exist before the MRCA of the current GII.4 variants, the occurrence of a population bottleneck may have left progeny virus of only one variant. The strains detected in the 1970s reported recently seem to confirm that multiple GII.4 variants existed before the Camberwell cluster arose [40]. Analyzing data of multiple NoV genotypes will provide a more detailed insight into the branching times of these different genotype clusters, and also in this case, including older sequences will be more elucidating.

### Possible shortcomings of this study

Ideally we would have used full genome sequences. However, for (GII.4) NoV these are only sparsely available. Instead, we showed that Bayesian coalescent demographic analyses of a large dataset containing very short sequences offered important and reliable insights into GII.4 variant dynamics. For the GII.4 NoV datasets presented here, the densely sampled but short polymerase sequences provided data that better defined the epidemic history than fewer longer capsid sequences. Perhaps additional to the limited size of the capsid dataset, strong selective pressure on the capsid protein confounded analysis of the capsid gene. We were also aware of the possibility that our capsid sequences dataset may have been biased in sampling. Sequencing of full capsid genes is not standard practice; the viruses of which sequences were available have all previously been selected by various researchers as ‘interesting enough to sequence’. Thus, relatively many sequences of strains belonging to the 1996 variant are present in the dataset, especially compared to strains belonging to the younger variants, 2004, 2006a and 2006b.

### Selective pressure shaped the population dynamics of NoV GII.4 variants

Our genealogical test clearly rejected neutral evolution for the NoV capsid dataset. Although P-values were somewhat higher using a Bayesian skyline plot model in the posterior predictive simulation compared to more restricted demographic functions, we arrived at the same conclusions for all analyses. This demonstrates that the neutrality test is not overly sensitive to the demographic detail in the analysis. Nevertheless, through the advances made here, we demonstrate that this test can now be performed under any complex demographic scenario, a generalization that may further promote its use. To investigate the selective forces in more detail, we fitted different evolutionary models to identify sites in the capsid under selective pressure. We examined an extension of previously performed dN/dS analyses [23], [40] to detect positively selected sites involved in population level selection, avoiding the effect of within host evolution. Eight positively selected codons were identified at *p*<0.05 with the iFEL approach. Of these sites, four (352, 372, 395 and 407) are located in the protruding regions of the protein. These sites have also been identified previously as ‘informative’ sites (at least two shared an identical amino acid mutation in the alignment) [20]. Amino acid 395, that was also detected as directionally evolving, is located in a surface exposed loop of the capsid protein, that is part of a variable site of carbohydrate interaction (amino acids 393-394-395) that has been identified by a number of studies as a locus for ligand binding and specificity [23], [52], [53]. Codon 394, located in this same domain, is part of an intricate network of co-evolving positions, also containing codons 297, 368 and 372. Amino acid 297 is part of a site identified by Allen *et al*, (296-297-298) [52], predicted as another of two host ligand binding sites. This particular site was not identified by Cao *et al.* [53] who performed co-crystallization assays with P-particle dimers and A and B-trisaccharides, the host ligands of NoV. It is structurally close to amino acids 368 and 372, on top of the protein, flanking the ligand binding pockets, and to 294, under directional evolution, which is located on the outside of the 296–298 loop, relative to the binding pocket.

Lindesmith *et al.* also identified sites under positive selection, using fewer sequences. They used three different methods, single likelihood ancestor counting (SLAC), fixed effects likelihood (FEL) and random effects likelihood (REL), under the Tamura-Nei model of evolution. More codons under positive selection were identified in this study, but less strict nominal significance values were used. Bok *et al.* [40] applied SLAC analyses for detection of positive selection and found six amino acids under positive selection. We chose to identify sites under selective pressure for internal branches in a phylogeny using iFEL because external branches are prone to deleterious mutational load. Such mutations are expected to be young and more likely fall on the external branches of a population-level phylogeny [54], where they can confound the identification of positively selected sites. To avoid this adverse effect we focused on internal branches only, on which advantageous mutations are more likely to fall.

Codon 333 was previously identified as an informative site [20] and our analyses found it to be under directional selection. It is located in the hydrophobic part of the P-dimer interface, just below the carbohydrate binding site described by Tan *et al.* [55], facing its counterpart in the other protomer of the same dimer (distance 4 Å) [53]. Changes in this amino acid are not likely to be involved in antigenic change but more likely structural compensation for other mutations.

### Positive selection is not only detected for sites involved in immune escape; secondary RNA structures are important for efficient RNA translation

Codons 6 and especially 9 have consistently been identified as sites under strong selective pressure, and involved in defining the distinction between variants, not only in this study but in others as well [23], [56]. Given their positions at the N-terminus of the protein, inside the shell, they seemed unlikely to have been under selective pressure through antibody recognition. This notion led us to investigate the RNA encoding the 5′end of ORF2. The nucleotide sequence upstream of codon 9 (nucleotide 26) is strongly conserved among all NoV genotypes, and a highly similar sequence is found at the 5′end of ORF1 in NoVs. Mutations have rarely been detected here, apart from at nucleotide 26, and at nucleotide 17 (codon 6). We propose that secondary structure predictions of these RNA regions provide an explanation for this pattern (Figure 5). Highly conserved stem-loop structures create the circumstances necessary for translation initiation of ORF1 and ORF2. Nucleotides A, C and G at position 26 all result in almost identical structures, in which 7 nucleotides from the start of ORF2, or 11 when including the 4 nucleotides upstream from the AUG, are left free. When theoretically a U/T is inserted here the predicted structure changes, resulting in a diminished length of the free nucleotide strand of 5 nucleotides. We are unaware of sequences with nucleotide T at position 26 present either in our or the public databases (data not shown), leading us to believe that a length of at least 7 unpaired nucleotides counting from the first AUG is necessary for efficient RNA translation from the subgenomic RNA. Thus, while normal capsids could be formed from strains with (silent) mutations in this area, the replication process of the virus may be disrupted by altering the secondary RNA structures. This theory is further supported by the observation that no other synonymous or non-synonymous mutations are found in the 9^th^ codon, e.g. at the third nucleotide position, nor at other nucleotides in this genomic area, apart from the previously mentioned nucleotide 17, in an RNA loop. Tentative analyses of other NoV genotypes demonstrated that the 5′ region is equally conserved within the different genotypes and yielded similar secondary RNA structures, which allow for point mutations in the loops of the structures (data not shown). To further substantiate this hypothesis site-directed mutagenesis studies are required, which go beyond the scope of this study.

### Identifying future new GII.4 variants

All of the substitutions described above were associated with at least one variant transition; they appear at branches that give rise to new variants (Figure 5 and Figures S2, S3A and S3B). This indicates that these mutations include the molecular determinants of cluster replacement. Previously identified antigenic sites [23], [52] did not enable distinction between all the different GII.4 variants (e.g. considering amino acids 296–298 and 393–395, the 2006b and 2007 variants that are clearly phylogenetically distinct, share identical amino acids).

When amino acids 6, 9, 294, 333, 352, 368, 372, 407 and 534, identified here as under positive, directional or co-evolutionary pressure, are added to these six amino acids, all currently identified GII.4 variants (excluding the Bristol and Camberwell strains, that circulated before the 1996 variant) are separated by at least two amino acid differences (Figure S4). Thus, specific analysis of these sites will aid early recognition of novel variants in the future. The two most recent distinct GII.4 variants, that have both been detected throughout the world in both 2008 and 2009, albeit at low prevalence, the 2007- and 2008-variants, are identical to the still dominant 2006b variant in amino acids 296–298, that were identified by Allen *et al*, and 2007 is also identical in site 393–395, whereas the 2008 variant has two substitutions on those sites (2006b: STT, 2008: D/NTA). Thus, considering the sites listed above, the 2008 variant would have the best chance of becoming the next dominant strain, whereas, when considering the full capsid sequence, the 2008 variant is more similar to the 2006b variant than is the 2007 variant.

Using Bayesian phylodynamic techniques we showed that since 2002 the number of GII.4 infections has experienced expansion dynamics. Additionally we further substantiated the evidence for signature sites for variant transition, which may aid in the early recognition of potential new epidemic variants, although we stress that examining pre-defined amino acids does not enable certain identification of GII.4 variants, for which full capsid sequences should be determined. We showed that it is important to select the genomic region to analyze by phylodynamic, coalescent methods with care, and our different datasets illustrated that for the phylodynamic analysis of pathogens undergoing repeated selective bottlenecks a considerable sampling density through time is required.

## Materials and Methods

### Datasets and data preparation

We compiled two different NoV GII.4 datasets. First, partial polymerase gene sequences with known detection month and year were collected. These sequences, encoding a short genomic region commonly referred to as Region A, have been collected for genotyping purposes, as an essential part of the ongoing surveillance practice in institutions around the world [22], [33]. This dataset includes sequences from participants of the Foodborne Viruses in Europe (www.fbve.nl) and of the Noronet (www.noronet.nl) networks, the contributing institutions of which are listed in the acknowledgements. Sequences of sufficient length (i.e. covering at least the final region) were included, generating a dataset of 1383 taxa, 247 nt in length. Strains originated from systematic surveillance collections, and form the best representative reflection of the circulating strains currently available.

Second, complete capsid sequences of GII.4 NoV strains with known sampling date were collected. This resulted in a dataset of 194 taxa, 1623 nt long. To allow comparison of results from capsid based versus polymerase based analysis, a set of 172 partial polymerase gene sequences matching the sequences in the capsids dataset (identical variant typing and similar detection dates) were selected from the total polymerase dataset. The 2003Asia variant was excluded from this mirror-dataset, since it was identified as recombinant and ORF1 does not belong to the GII.4 genotype.

Details on the nature of the strains comprised by the two generated datasets are provided in the Supporting Materials Table S1. The distribution of the sampling dates of the included sequences is depicted in Figure S5.

For recombination analyses, a set comprising 20 sequences, two of each GII.4 variant, spanning the genome region between Region A, in ORF1, and the complete capsid sequence was collected.

Sequences were aligned using the Clustal W algorithm implemented in Bioedit (version 7.0.9.0) and edited where necessary. Sequence alignments can be obtained from the authors on request.

### Recombination analysis

Recombination within the genomic area under study invalidates the use of phylogenetic approaches. Therefore, we checked for possible recombination signal by analyzing 20 sequences (two for each variant) spanning Region A through the complete capsid protein (2404 nt). Different evolutionary histories across this genome region were inferred using the genetic algorithm for recombination detection (GARD) [57] and specific recombinants were identified using a modified VisRD algorithm [58] and using RDP3 [59]. In addition, we used the Phi test, shown to perform well under strong population growth and to be able to distinguish recurrent mutations from recombination events, to identify recombination signal in the NoV alignments [36].

### Time-measured phylodynamic analyses and associated neutrality test

Evolutionary dynamics were estimated using a Bayesian Markov chain Monte Carlo (MCMC) approach implemented in BEAST (BEAST version 1.4.7 [60]). BEAST MCMC analysis estimates marginal posterior distributions for every parameter in a full probabilistic model comprising the timed evolutionary history, based on the incorporation of sampling times in a molecular clock model, the substitution process and demographic history. We used the GTR+I+Γ_4_ model of substitution and the uncorrelated lognormal relaxed clock model to accommodate variation in substitution rates among different branches [61].

To test selective neutrality of GII.4 molecular evolution, we adopted the genealogical framework presented by Drummond and Suchard [37]. This involves the full model-based Bayesian analysis to obtain a posterior distribution of trees, genealogical summary statistics, and posterior predictive simulation to detect departures from the neutral expectations for these statistics. We employed the genealogical Fu and Li statistic (*D_F_*), which compares the length of terminal branches to the total length of the coalescent genealogy. Strongly negative values for this statistic indicate terminal branch lengths being larger than expected, which reflects an excess of slightly deleterious mutations on these branches. This statistic has proven to be most sensitive in uncovering non-neutral evolution, and has for example rejected neutrality for human IAV hemagglutinin genes [37]. Posterior predictive simulation is performed according to the same demographic model as used in to obtain the posterior tree distribution. To evaluate the impact of large demographic trends, we compared analyses using both constant and exponential growth population size priors. To further investigate the impact of demographic detail on the neutrality test we extended the simulation procedure to highly parametric demographic models, including piecewise constant demographic functions that define a Bayesian skyline plot model. We validated the simulation procedure by comparing the reconstructed Bayesian skyline plot from the trees generated by posterior predictive simulation with the Bayesian skyline plot inferred from the sequence data, which yielded consistent results.

To reconstruct the NoV GII.4 demographic history in more detail, we employed the Bayesian skyline plot (BSP) model, which generates piecewise constant population size trajectories [62]. In this coalescent setting, demography is measured as the product of effective population size (N_e_) and generation time (τ), N_e_ τ, through time. To obtain a detailed measurement of NoV GII.4 diversity through time, given the large dataset, we specified 40 groups in the piecewise constant population size function. All chains were run sufficiently long to achieve stationarity after burn-in, as checked using TRACER (http://tree.bio.ed.ac.uk/software/tracer/).

Additionally, the polymerase dataset was split up into separate subsets, each comprising all available sequences from a major GII.4 variant, and these were analyzed individually using the same model and settings as was used for the whole polymerase dataset.

To examine how different sampling densities through time can impact our demographic estimates, we performed an additional analysis of a subset of the polymerase data, containing sequences selected to best mirror the sequences in the capsid dataset, using the same specifications as described above.

### Analyses of molecular adaptation

#### Codon substitution analyses

Selective pressure in the capsid genes was analyzed using the nonsynonymous/synonymous rate ratio (*dN/dS*) in a codon model framework (reviewed in [63]). Probabilistic models of codon substitution can be used to identify various types of selection pressures in the evolutionary history of a gene. Diversifying positive selection is characterized by *dN/dS*>1, which indicates that (adaptive) nonsynonymous substitutions have accumulated faster than synonymous substitutions. We considered a generalization of the random effects *Dual* model [64], where synonymous and non-synonymous rates are drawn from independent general discrete distributions. In this study, we fitted a general bivariate discrete distribution (GBDD) of substitution rates to the data using maximum likelihood in HyPhy [41], [65]. A GBDD on *D* rates estimates 3*D*-2 parameters: a pair of rates (synonymous and non-synonymous) and a weight for each class. Two constraints that must be satisfied by the parameters are: the weights must sum to one, and the mean for synonymous rates must also be one to avoid confounding the mean of substitution rates and the length of the tree (see [64] for details). This distribution allows dN and dS to co-vary, by directly estimating the values for dS and dN for *D* rate classes, instead of assuming that they are selected independently. The value of *D* was determined by a step-up procedure on *D* (starting with *D* = 1), where the fit of models with *D* and *D*+1 rate classes was compared using small sample AIC. Posterior distributions of substitution rate parameters were approximated using sampling importance resampling. We identified sites under diversifying or positive selection using an empirical Bayes procedure. We chose an empirical Bayes Factor cut-off of 20 to classify positively selected sites [64]. Additionally we carried out a selection analysis using a random effects model (REL), and a fixed effects likelihood (FEL) approach [66] to confirm the predictions of the REL method and performed dataset-specific simulations [67] on internal braches, as considering internal branches is more relevant than the analysis of terminal branches in population level selection [42], to estimate the operating characteristics of the site-specific likelihood ratio test.

#### Directional evolution analyses

To detect directional selection we used the DEPS test that identifies statistically significant shifts in amino acid residue frequencies over the tree and/or an unusually large number of substitutions towards a particular residue in a maximum likelihood context [38]. DEPS tests whether the amino acid substitution rate towards a particular residue as estimated using a directionally biased model is significantly different from baseline, reversible substitution rates. For this procedure, we used the amino acid substitution rates estimated under the general reversible protein model (REV) for the baseline and directionally biased models and employed the rooted maximum clade credibility from the BEAST analysis.

#### Epistatic effects, or co-evolutionary analyses of amino acids

Co-evolving sites, showing evidence for concurrent amino acid substitutions, were identified using Bayesian graphical models (BGM) in a phylogenetic framework [68]. Briefly, this method infers the history of substitution events using a codon-based maximum likelihood phylogenetic approach. Correlated patterns of nonsynonymous substitutions are subsequently identified using BGMs and an order-MCMC algorithm. Two modes of analyses were performed allowing either a single or two co-dependencies per site. We reported sites identified with P>0.9 by either mode.

### RNA secondary structure predictions

Secondary structure predictions of the 5′end of the ORF2 encoding RNA were generated using the web based RNA Fold Webserver (http://rna.tbi.univie.ac.at/cgi-bin/RNAfold.cgi) [69].

## Supporting Information

Figure S1 Comparison of Bayesian Skyline Plots of the polymerase dataset, the capsid dataset, and the dataset comprised of partial polymerase sequences matching the sequences in the capsid dataset.
(0.25 MB TIF)

Figure S2 Sites identified to be under directional selection by DEPS analysis. Sites are depicted by MCC trees colored for which amino acid was present on each branch.
(0.25 MB TIF)

Figures S3 Co-evolving sites. S3A) Sites 238 and 504. S3B) Sites 231 and 509.
(0.28 MB TIF)

Figure S4 Minimum Spanning Tree (MST) of amino acids 6, 9, 294, 296-297-298, 333, 352, 368, 372, 393-394-395, 407, 534. Thick lines represent distances of 1 or 2 amino acids, thin lines 3 or more. An MST connects all samples in such a manner that the summed distance between all samples or branches is minimized. Different colors indicate the different variants. Different variants are separated by at least 2 amino acids.
(0.52 MB TIF)

Figures S5 Detection dates of NoV GII.4 strains included in the study. S5A) The polymerase sequence detection dates. S5B) The capsid sequence detection dates.
(0.18 MB TIF)

Table S1 Background information on strains comprised by the two datasets. NA: Not Assigned, C'well: Camberwell.
(0.09 MB DOCX)
