# Supplementary figures and images for: Phylodynamic Reconstruction Reveals Norovirus GII.4 Epidemic Expansions and their Molecular Determinants
Source: PLoS Pathog. 2010 May 6;6(5):e1000884. doi: 10.1371/journal.ppat.1000884 (PMC2865530; doi:10.1371/journal.ppat.1000884)

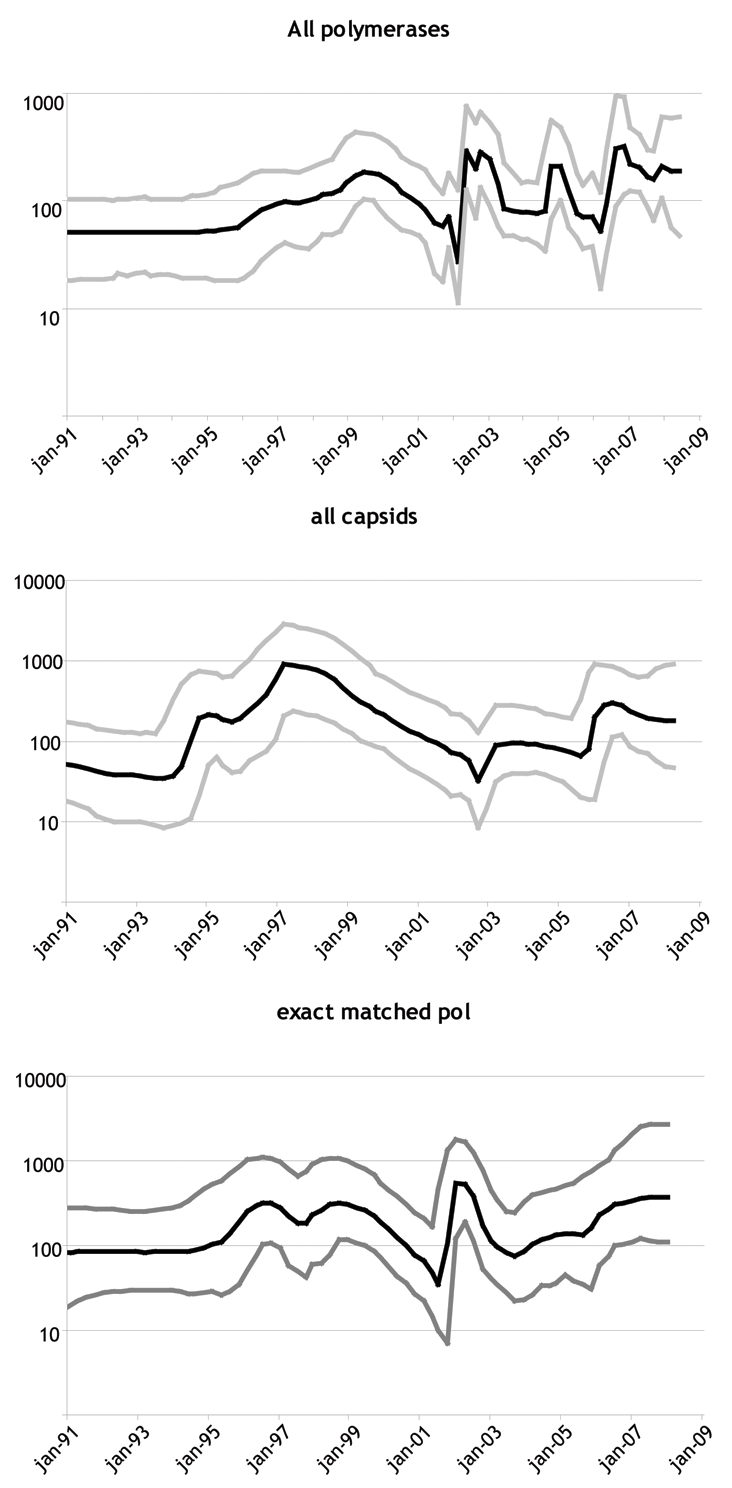

Supplement: Figure S1 — Comparison of Bayesian Skyline Plots of the polymerase dataset, the capsid dataset, and the dataset comprised of partial polymerase sequences matching the sequences in the capsid dataset. (0.25 MB TIF) [file ppat.1000884.s001.tif]

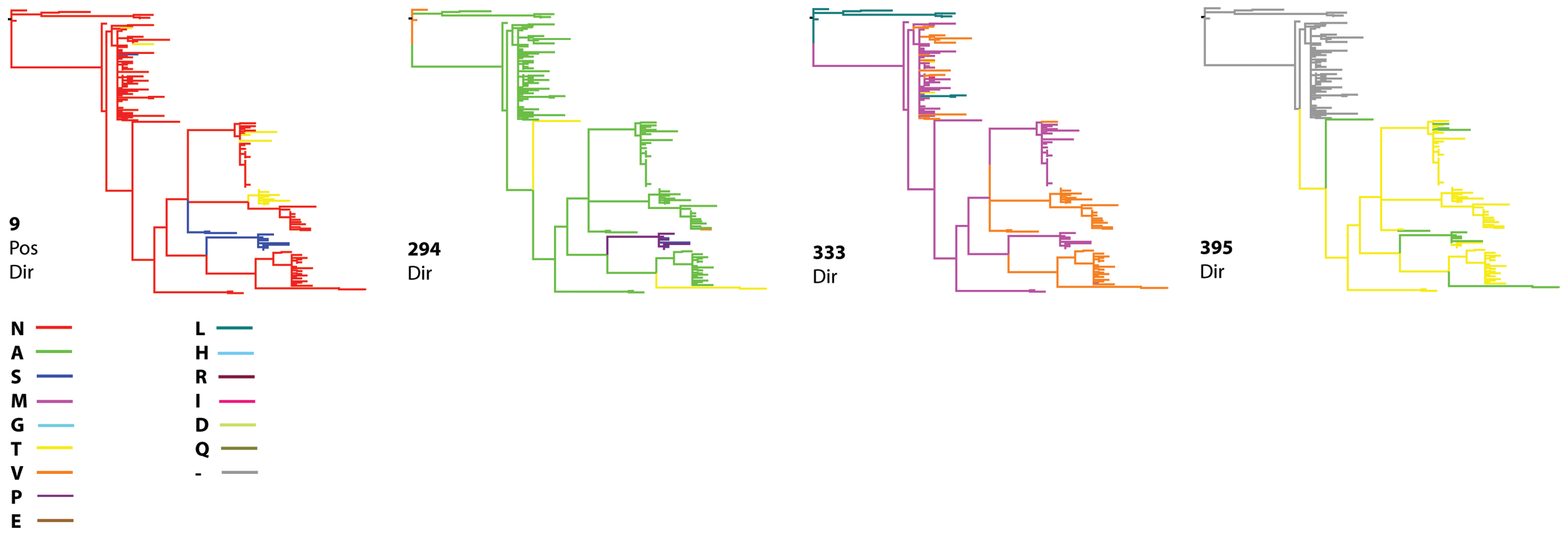

Supplement: Figure S2 — Sites identified to be under directional selection by DEPS analysis. Sites are depicted by MCC trees colored for which amino acid was present on each branch. (0.25 MB TIF) [file ppat.1000884.s002.tif]

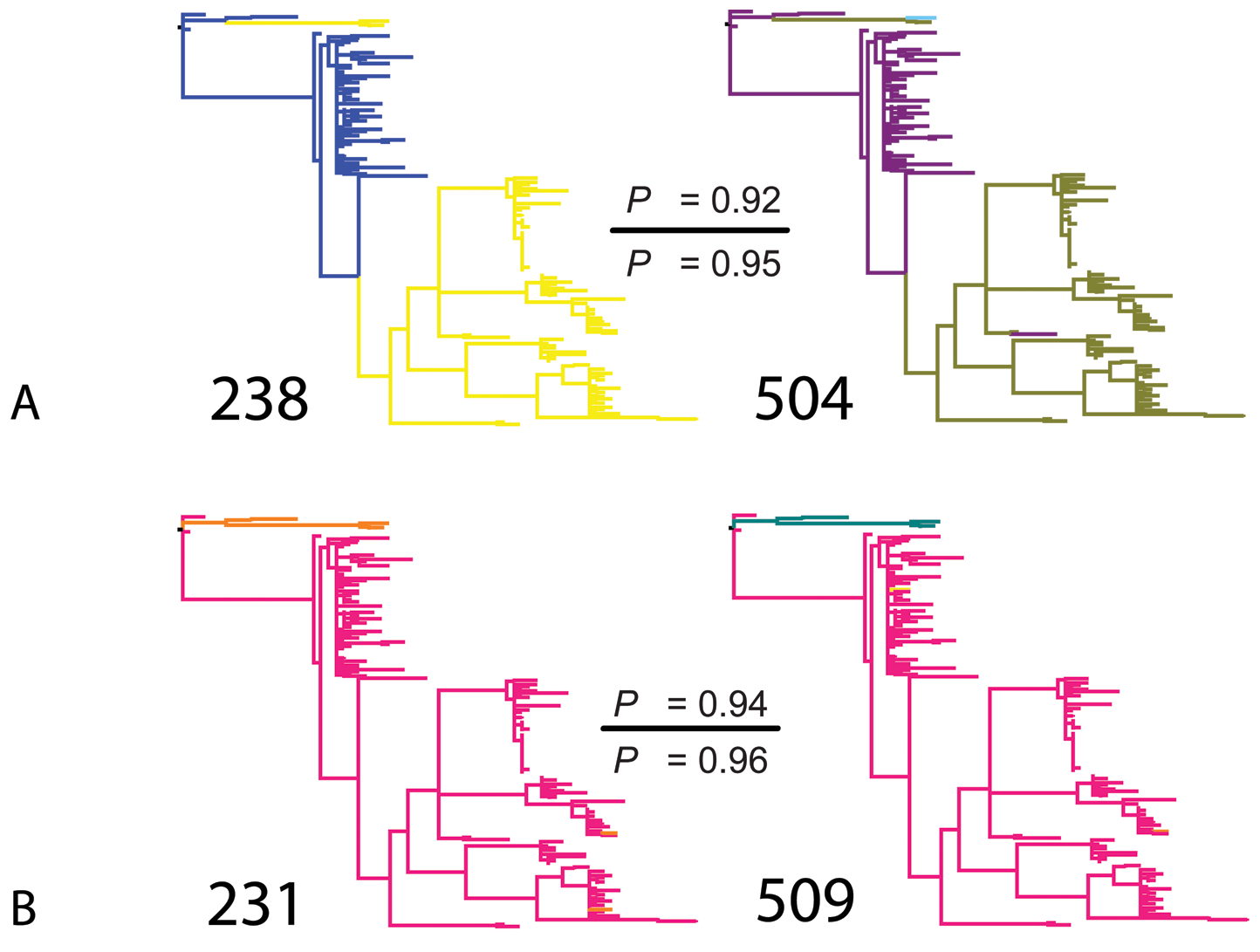

Supplement: Figures S3 — Co-evolving sites. S3A) Sites 238 and 504. S3B) Sites 231 and 509. (0.28 MB TIF) [file ppat.1000884.s003.tif]

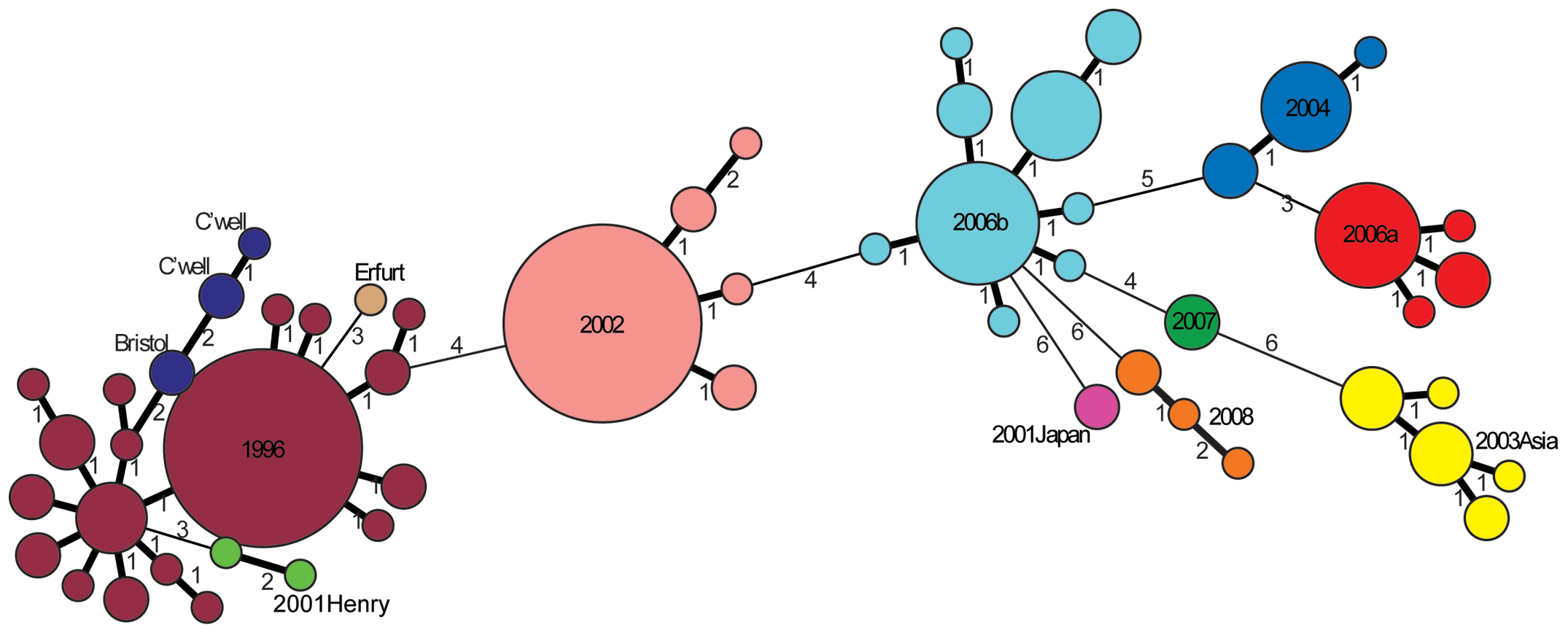

Supplement: Figure S4 — Minimum Spanning Tree (MST) of amino acids 6, 9, 294, 296-297-298, 333, 352, 368, 372, 393-394-395, 407, 534. Thick lines represent distances of 1 or 2 amino acids, thin lines 3 or more. An MST connects all samples in such a manner that the summed distance between all samples or branches is minimized. Different colors indicate the different variants. Different variants are separated by at least 2 amino acids. (0.52 MB TIF) [file ppat.1000884.s004.tif]

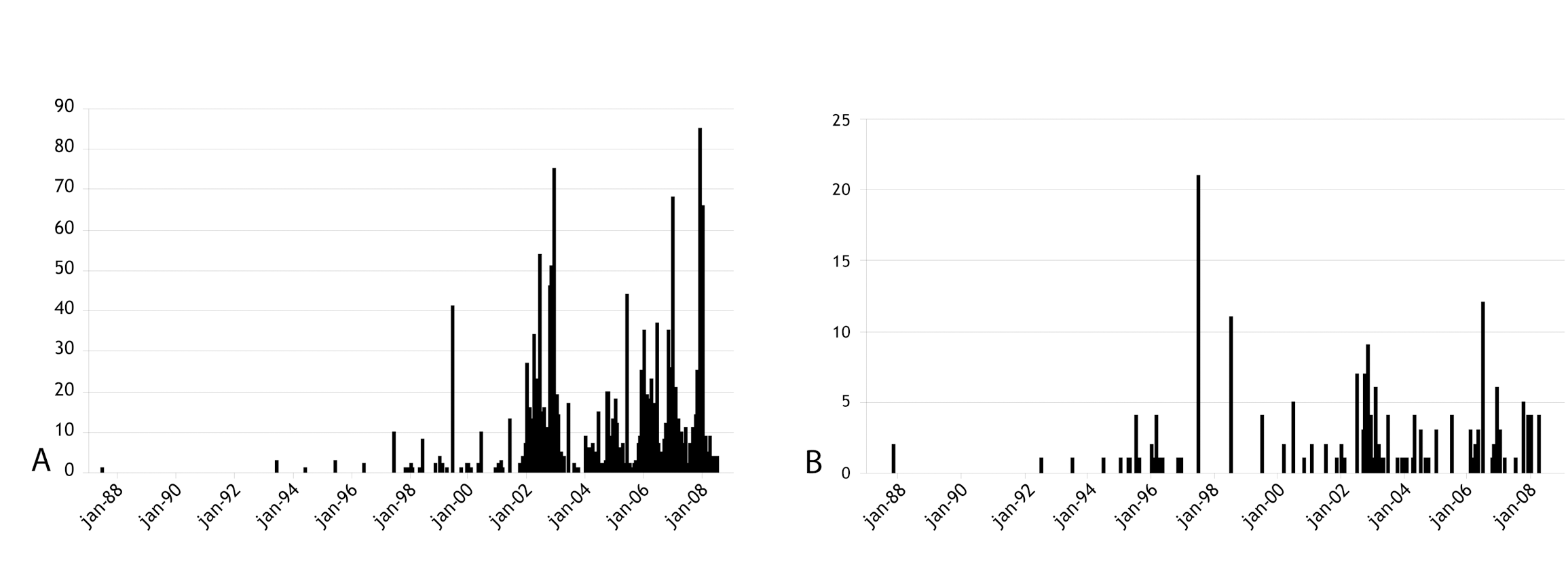

Supplement: Figures S5 — Detection dates of NoV GII.4 strains included in the study. S5A) The polymerase sequence detection dates. S5B) The capsid sequence detection dates. (0.18 MB TIF) [file ppat.1000884.s005.tif]
