# Supplementary material for: Phylodynamic Reconstruction Reveals Norovirus GII.4 Epidemic Expansions and their Molecular Determinants
Source: PLoS Pathog. 2010 May 6;6(5):e1000884. doi: 10.1371/journal.ppat.1000884 (PMC2865530; doi:10.1371/journal.ppat.1000884)
Supplement: Table S1 — Background information on strains comprised by the two datasets. NA: Not Assigned, C'well: Camberwell. (0.09 MB DOCX) [file ppat.1000884.s006.docx]

Table S1. Background information on strains comprised by the two datasets. NA: Not Assigned, C’well: Camberwell

| ***Capsid dataset*** |  |  |  |  |  |  |  |  |  |  |  |  |
| --- | --- | --- | --- | --- | --- | --- | --- | --- | --- | --- | --- | --- |
| **Geographical origin** | **Oceania** | **North America** | **South America** | **Asia** | **Europe** | **Africa** | **Total** |  |  |  |  |  |
| Strains (%) | 7 (3.61) | 22 (11.34) | 1 (0.52) | 29 (14.95) | 135 (69.59) | 0 (0) | 194 (100) |  |  |  |  |  |
| **Variant** | **1996** | **2002** | **2004** | **2006a** | **2006b** | **2001Japan** | **2002/CN**  **/ Henry** | **2003Asia** | **Bristol** | **C’well** | **NA** | **Total** |
| Strains (%) | 67 (34.54) | 45 (23.20) | 12 (6.19) | 17 (8.76) | 24 (12.37) | 4 (2.06) | 2 (1.03) | 12 (6.19) | 2 (1.03) | 3 (1.55) | 6 (3.09) | 194 (100) |
| ***Polymerase dataset*** |  |  |  |  |  |  |  |  |  |  |  |  |
| **Geographical origin** | **Oceania** | **North America** | **South America** | **Asia** | **Europe** | **Africa** | **Total** |  |  |  |  |  |
| Strains (%) | 3 (0.22) | 6 (0.43) | 1 (0.07) | 60 (4.34) | 1312 (94.87) | 1 (0.07) | 1383 (100) |  |  |  |  |  |
| **Variant** | **1996** | **2002** | **2004** | **2006a** | **2006b** | **2001Japan** | **2002/CN**  **/ Henry** | **2003Asia** | **Bristol** | **C’well** | **NA** | **Total** |
| Strains (%) | 175 (12.65) | 433 (31.31) | 214 (15.47) | 159 (11.50) | 377 (27.26) | 16 (1.16) | 1 (0.07) | Not Applicable | 1 (0.07) | 5 (0.36) | 2 (0.14) | 1383 (100) |
